# Link between the albumin-corrected anion gap and 28 day all‑cause mortality among patients with sepsis complicated with chronic heart failure: A retrospective analysis using the eICU Collaborative Research Database

**DOI:** 10.1371/journal.pone.0337973

**Published:** 2025-12-16

**Authors:** Chen Zhang, Ali Ma, Juan Ma, Peng Wu, Huiyan Ma, Xueping Ma, Ning Yan

**Affiliations:** 1 First Clinical College, Ningxia Medical University, Yinchuan, People’s Republic of China; 2 Heart Centre and Department of Cardiovascular Diseases, General Hospital of Ningxia Medical University, Yinchuan, People’s Republic of China; 3 Department of Geriatrics and Special Needs Medicine, General Hospital of Ningxia Medical University, Yinchuan, People’s Republic of China; 4 Institute of Medical Sciences, General Hospital of Ningxia Medical University, Yinchuan, People’s Republic of China; University of Rijeka Faculty of Health Studies: Sveuciliste u Rijeci Fakultet zdravstvenih studija, CROATIA

## Abstract

**Background:**

These two conditions, namely metabolic acidosis and hypoproteinemia, are prevalently observed in patients within intensive care units (ICU), particularly those with sepsis complicated with chronic heart failure. Nevertheless, the impact of the Albumin-Corrected Anion Gap (ACAG), an indicator reflecting the above conditions, on such patient mortality requires further investigation. This retrospective cohort study analyzed the significance of ACAG levels in forecasting 28-day all-cause mortality among these patients admitted to ICU.

**Methods:**

This was observational cohort study on eICU Collaborative Research Database (eICU-CRD) that included in participants with sepsis complicated with chronic heart failure. In the study, we applied several methods such as multivariate Cox regression models and smooth curve fitting plots combined with Kaplan-Meier analysis to investigate how ACAG is correlated with 28 day all‑cause mortality. To explore the results’ stability, subgroup analysis was performed and a forest plot was plotted.

**Results:**

The final analysis included 713 eligible participants after rigorous screening procedures. The mean level of ACAG was (16.68 ± 5.20) mmol/l. The 28-day mortality rate was 13.60% (97/713) in our study. The multivariate Cox regression analysis revealed a significant association between ACAG (as a continuous variable) and 28-day all-cause mortality, unadjusted model (HR 1.07, 95% CI 1.04–1.11, p < 0.0001), adjusted model 1 (HR 1.08, 95% CI 1.04–1,12, *p* < 0.0001), adjusted model II (HR, 1.08 (1.03,1.13), *p* < 0.001). After adjusting for all confounding factors (listed in the Model II), the smoothing curves showed a linear relationship. Mortality in such patients gradually increased with the increase of ACAG according to Kaplan-Meier analysis. Subgroup analysis illustrates the stability of the link between ACAG and 28-day mortality in participants with sepsis complicated with chronic heart failure across various subgroups.

**Conclusions:**

After adjusting for confounding factors, elevated ACAG is positively linked with increased 28-day mortality in patients with sepsis complicated with chronic heart failure.

## 1. Introduction

Sepsis, referred to as a life-threatening organ dysfunction attributable to infection, poses a significant global health challenge, affecting over 30 million individuals annually and leading to mortality rates exceeding 20% among critically ill populations [1]. Studies have shown that individuals who experience sepsis possess a twofold greater risk of mortality for up to five years post-event compared to those who do not develop sepsis [2,3]. The prognosis for septic patients, particularly those with underlying comorbidities, remains grim. Among these comorbidities, chronic heart failure (CHF) represents a critical condition that significantly alters the pathophysiological landscape and amplifies the risk of mortality. The complexity of sepsis is further compounded by the presence of chronic heart failure (CHF), a common comorbidity that exacerbates patient outcomes due to impaired cardiac function and systemic hypoperfusion [4]. Furthermore, the presence of comorbidities such as CHF can significantly worsen outcomes, as these patients are more susceptible to complications related to cardiovascular instability and metabolic derangements [5,6].

Acid-base imbalance and malnutrition are closely associated with disease prognosis and may significantly impact patient outcomes. The anion gap (AG) serves as a crucial indicator of acid-base imbalance and is significantly influenced by serum albumin levels. The albumin corrected anion gap (ACAG) adjusts for hypoalbuminemia, thereby enhancing diagnostic accuracy in critically ill patients. Research has established a link between ACAG and mortality in sepsis as well as cardiovascular diseases separately [2,7]. However, the specific role of ACAG in the context of sepsis complicated by CHF remains largely unexplored. The comorbidity of sepsis and congestive heart failure is a fatal combination. The two exacerbate each other through complex pathophysiological mechanisms, leading to difficult treatment and an extremely high mortality rate. Sepsis and heart failure can form a fatal vicious cycle where “1+1 is far greater than 2”. The immense pressure of sepsis can cause an already fragile heart to collapse instantly, and a weak heart in turn makes the treatment of sepsis a dilemma (failure to receive fluid replacement leads to shock, and fluid replacement leads to heart failure), ultimately greatly increasing the risk of organ failure and death. Previous studies have lacked evidence to investigate the relationship between ACAG and 28-day mortality in such populations. Therefore, a significant knowledge gap persists. This gap in the literature necessitates further investigation into the link between ACAG and 28-day all-cause mortality in this high-risk population, as understanding this correlation could provide noteworthy insights into patient prognosis and management strategies.

Under this Introduction, we hypothesized that higher serum concentration of ACAG in patients with sepsis complicated with chronic heart failure might be correlated with more serious homeostatic imbalance and, therefore, worse clinical results. The primary aim of this investigation was to examine the link between ACAG and 28-day all-cause mortality in critically ill patients diagnosed with sepsis complicated with chronic heart failure during ICU hospitalization.

## 2. Materials and methods

### 2.1. Data source

This retrospective cohort analysis utilized data obtained from the eICU Collaborative Research Database (eICU-CRD), a publicly accessible, multicenter repository containing granular clinical records for more than 200,000 intensive care unit admissions across U.S. hospitals monitored by the eICU program [8]. Spanning the years 2014–2015, the dataset was automatically captured and archived via the Philips Healthcare eICU platform before being made available for research purposes [8]. Previous investigations have demonstrated the database’s utility in observational studies addressing diverse critical care challenge [9–13]. Access to the database requires fulfilling eligibility criteria mandated by the PhysioNet Review Board, including successful completion of a standardized assessment and formal certification aligned with their data governance protocols [14]. This resource operates in compliance with the Health Insurance Portability and Accountability Act (HIPAA) Safe Harbor provision [14]. Data extraction was performed by researcher Zhang following approval of access, which was contingent upon completion of the Collaborative Institutional Training Initiative (CITI) program“Data or Specimens Only Research”training module (certification number: 64522028).

### 2.2. Ethics approval and consent to participate

This retrospective study utilized deidentified data from the eICU Collaborative Research Database (eICU-CRD), compliant with the data usage agreement (our record ID: 64522028) by the PhysioNet review committee and compliant with HIPAA Safe Harbor provisions by Privacert (Cambridge, MA). This study involved secondary analysis of anonymized clinical data obtained from a publicly accessible repository, precluding the need for formal ethics review by either the Massachusetts Institute of Technology’s Institutional Review Board or local ethics committees due to its non-interventional design and compliance with Safe Harbor deidentification standards. Informed consent requirements were waived as no direct patient contact occurred. The protocol adhered rigorously to the ethical principles outlined in the Declaration of Helsinki, with all analytical procedures aligning with international research guidelines and institutional regulatory frameworks.

### 2.3. Study population

**Fig 1:**
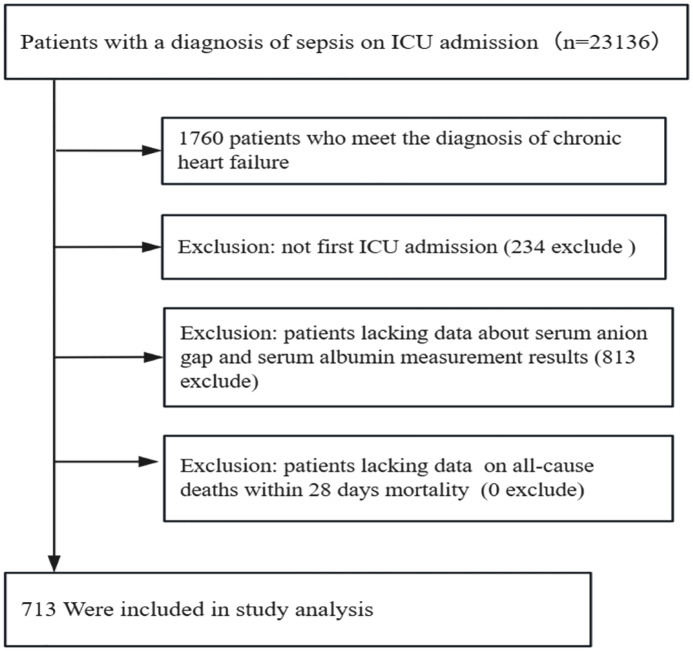
Flow chart of study population. The final analysis included 713 eligible participants after rigorous screening procedures.

Our target study population encompassed participants with sepsis complicated with chronic heart failure. The diagnostic criteria for sepsis and chronic heart failure are outlined below. Firstly, the Sequential Organ Failure Assessment (SOFA) score serves as a clinical scoring system designed to evaluate and quantify the extent of organ dysfunction or failure in hospitalized patients, specifically developed for monitoring disease progression and treatment responses in critically ill populations [15]. The present study employed the SOFA scoring system in strict accordance with international critical care guidelines. Study enrollment was restricted to individuals fulfilling Sepsis-3 criteria, characterized by an acute change of at least two points in the SOFA score as a result of infection [16]. Besides, the diagnosis of chronic heart failure required comprehensive evaluation integrating symptomatic manifestations, Doppler echocardiographic evidence of cardiac remodeling (including hemodynamic alterations such as reduced left ventricular ejection fraction <50%), and serum biomarker for brain natriuretic peptide (BNP)or N-terminal pro-brain natriuretic peptide (NT-proBNP) [17,18]. In the present study, we include that participants with a diagnosis of sepsis on ICU admission (n = 23136). Among these people, 1760 patients who meet the diagnosis of chronic heart failure. The following exclusion criteria were applied:(1) not first ICU admission (234 exclude); (2) patients lacking data about serum anion gap and serum albumin measurement results (813 exclude) and (3) patients lacking data on all-cause deaths within 28 days mortality (0 exclude) (Fig 1).

### 2.4. Variables

The eICU database includes demographic records, physiological indicators of bedside monitors, diagnosis via the International Classification of Diseases, 9^th^ Edition, Clinical Modification (ICD-9-CM) codes, and other laboratory data obtained during routine medical care [14]. The eICU-CRD was utilized to gather data from all participants within the initial 24 hours of hospital admission [14]. All physiological and biochemical parameters were evaluated using the initial daily measurement obtained within 24-hour cycles, with strict adherence to the principle of temporal precedence in clinical data acquisition. The demographic characteristics, physiological parameters, laboratory parameters, previous history, severity scoring systems, and outcomes of the included participants were collected. The demographic characteristics, including age, gender, height, weight, and body mass index (Body Mass index (BMI) = weight (kg)/height (m) squared), these bits of data were gotten respectively from both the tables of patient and apachePatientResult tables. The physiological parameters that are like temperature measured in Celsius, respiratory rate, heart rate (HR), mean arterial pressure (MAP), were pulled right out from the apacheApsVar table. The laboratory parameters of PH value, blood urea nitrogen (BUN), glucose, serum creatinine, anion gap (AG), serum potassium, albumin, white blood cell count, and red blood cell count were obtained from the laboratory tables. In particular, ACAG refers to the albumin-corrected anion gap, which is calculated using a specific algorithm based on the serum albumin level and the anion gap derived from laboratory measurements. Previous histories including acute myocardial infarction (AMI), diabetes, and arrhythmia were collected from the APACHE IV score. Severity level at admission was measured by the Acute Physiology Score III and Apache IV score (APS III, APACHE IV).

### 2.5. Definition of ACAG and outcome

The outcome of this study was 28-day all-cause mortality, defined as deaths from any cause occurring within the first 28 days after ICU admission. This study implemented a 28-day observation period to systematically monitor the survival status of patients. According to prior research, the ACAG is computed as follows: ACAG (mmol/l) = [4.4-observed albumin (g/dl)] * 2.5 + observed AG [19].

### 2.6. Statistical analysis

Normally distributed continuous variables should be expressed in the format of mean ± standard deviation (SD). Continuous variables with non-normal distributions are presented using the median and interquartile range. Categorical variables should be presented as counts and percentages. Comparative analyses between groups were conducted using one-way ANOVA for continuous variables and Chi-square tests for categorical variables, with statistical methods appropriately applied to their corresponding data types. The study cohort was divided into three groups based on ACAG tertile, with corresponding baseline characteristics comprehensively summarized in Table 1. After considering the previous research and clinical expertise, the multivariate Cox model was created to determine the effects of the three groups on 28-day all-cause mortality. Two models were constructed: Model 1 was adjusted for gender, age and BMI. Model 2 adjusted for age, sex, BMI, Temperature, Heart rate, MAP, Blood urea nitrogen, Glucose, Serum Creatinine, Serum potassium, Diabetes, AMI, Arrhythmia, acute Physiology Score III, Apache IV score. To investigate how ACAG is correlated with 28 day all‑cause mortality. To verify the linear relationship between ACAG and 28-day all-cause mortality, we adjusted for all confounder factors included in Model II and generated smooth curves as well as Kaplan-Meier survival curve. Stratified analyses were conducted based on age (< 74 years old or ≥ 74 years old), BMI (< 28.08 or ≥ 28.08), temperature (< 36.4 or ≥ 36.4), heart rate (< 109 or ≥ 109), MAP (< 56 or ≥ 56), BUN (< 35 or ≥ 35), glucose (< 133 or ≥ 133), diabetes, acute physiology score III, and Apache IV score. The forest plot was drawn according to the results of stratified analysis. The missing data are documented in the notes of Table 1. We employed multiple imputation, based on five replications and the chained equations approach implemented in the R MI procedure, to address missing data. we examined potential bias arising from the use of indicator variables for handling missing data by performing a multiple-imputation analysis. To evaluate the robustness of our results, sensitivity analyses were conducted. All the statistical analyses were performed using the EmpowerStats (www.empowerstats.com, X&Y solutions, Inc. Boston MA) and R software version 3.6.1 (http://www.r-project.org) [14].

**Table 1:** Baseline characteristic of participants (N = 713).

Variables	Total Population (n = 713)	ACAG Tertiles			*p* value
		T1(n = 235) 4.75-14.25	T2 (n = 240) 14.25-18.05	T3 (n = 238) 18.05-41.50	
**Demographic Characteristics**					
Age (years)	72.06 ± 13.03	73.74 ± 12.46	71.36 ± 13.74	71.11 ± 12.75	0.045
Sex (Male)	344 (48.25%)	108 (45.96%)	120 (50.00%)	116 (48.74%)	0.666
Body Mass Index (kg/m^2^)	30.08 ± 9.59	30.78 ± 10.50	29.49 ± 9.09	29.98 ± 9.12	0.645
**Physiological Parameters**					
Body Temperature (°C)	36.48 ± 1.22	36.58 ± 1.00	36.53 ± 1.14	36.32 ± 1.48	0.093
Respiratory Rate (breaths/min)	31.10 ± 13.98	30.28 ± 13.78	31.32 ± 14.22	31.69 ± 13.96	0.520
Heart Rate (beats/min)	107.56 ± 29.48	103.09 ± 30.14	109.49 ± 29.74	110.03 ± 28.16	0.009
Mean Arterial Pressure (mmHg)	78.25 ± 43.61	76.00 ± 39.74	81.30 ± 45.20	77.42 ± 45.61	0.104
**Laboratory Parameters**					
pH	7.34 ± 0.12	7.35 ± 0.11	7.36 ± 0.11	7.32 ± 0.14	0.084
Blood Urea Nitrogen (mg/dL)	42.43 ± 26.32	35.23 ± 19.45	40.40 ± 23.79	51.59 ± 31.57	<0.001
Glucose (mg/dL)	154.19 ± 81.10	143.14 ± 68.02	156.14 ± 75.65	163.17 ± 96.04	0.202
Serum Creatinine (mg/dL)	2.25 ± 1.72	1.63 ± 1.02	2.08 ± 1.37	3.02 ± 2.23	<0.001
Anion Gap(mmol/l)	12.38 ± 5.05	7.54 ± 2.22	11.84 ± 1.68	17.68 ± 4.18	<0.001
Serum Potassium (mmol/L)	4.31 ± 0.82	4.25 ± 0.76	4.23 ± 0.76	4.44 ± 0.91	0.023
Albumin(g/dl)	2.68 ± 0.57	2.86 ± 0,53	2.68 ± 0.55	2.50 ± 0.59	<0.001
White Blood Cell Count (×10⁹/L)	15.00 ± 9.30	13.65 ± 8.00	15.36 ± 9.97	16.01 ± 9.69	0.027
Red Blood Cell Count (M/mcl)	3.63 ± 0.75	3.58 ± 0.68	3.61 ± 0.74	3.68 ± 0.81	0.668
**Previous history**					
Previous Acute Myocardial Infarction	54 (7.57%)	15 (6.38%)	15 (6.25%)	24 (10.08%)	0.200
Previous Diabetes Mellitus	173 (24.26%)	45 (19.15%)	53 (22.08%)	75 (31.51%)	0.005
Previous Arrhythmia	248 (34.78%)	74 (31.49%)	79 (32.92%)	95 (39.92%)	0.119
**LOS (day)**	4.54 ± 5.50	4.04 ± 3.94	4.63 ± 6.34	4.93 ± 5.88	0.202
**Severity Scores**					
Acute Physiology Score III	59.27 ± 22.66	52.98 ± 21.19	59.32 ± 22.05	65.51 ± 23.04	<0.001
Apache IV score	75.29 ± 23.34	69.36 ± 22.69	75.36 ± 22.98	81.14 ± 22.96	<0.001
**Mortality**					
**Yes**	97 (13.60%)	18 (7.66%)	35 (14.58%)	44 (18.49%)	0.002
**No**	616 (86.40)	217 (92.34%)	205 (85.42%)	194 (81.51%)	

**Notes:** Data are expressed as the mean ± SD, median (interquartile range), or percentage. Among the 713 patients, the amount of missing values for the covariates were 19 (2.7%) for admission BMI, 42 (5.9%) for temperature, 6 (0.8%) for respiratory rate, 4 (0.6%) for heart rate, 4 (0.6%) for MAP, 452 (63.4%) for PH value, 8 (1.1%) for glucose, 1(0.1%) for serum creatinine, 27 (3.8%) for red blood cell count, 27 (3.8%) for white blood cell count, 120 (16.8%) for Apache IV score, 120 (16.8%) for Acute Physiology Score III. All individual missing values were deleted. p-value: For continuous variables, the Kruskal Wallis rank sum test is used. For categorical variables with a theoretical number<10, the Fisher’s exact probability test is used.

**Abbreviations:** ACAG, Albumin Corrected Anion Gap; BMI, body mass index; MAP, mean arterial pressure; ICU, intensive care unit, LOS (day), length of ICU stay (day).

## 3. Results

### 3.1. Baseline characteristics

The study population comprised 713 participants (mean age 72.06 ± 13.03 years; 48.25% male), whose comprehensive baseline demographic and clinical characteristics are detailed in Table 1. We conducted a comprehensive analysis of the study population through the tertiles of the ACAG. The enrolled participants were categorized into three groups by the tertiles of ACAG as follows: T1 group, 4.75–14.25 mmol/l; T2 group, 14.25–18.05 mmol/l; and T3 group, 18.10–41.50 mmol/l. Key findings revealed statistically significant differences across ACAG tertiles in several critical variables. Compared with subjects in T1 group (the lowest tertile of ACAG), subjects in T3 group (the highest tertile of ACAG) were younger and had Lower BMI at admission. Heart rate demonstrated a progressive increase across tertiles (*p *= 0.009), indicating potential physiological alterations. Laboratory parameters exhibited significant trends, particularly in BUN (*p *< 0.001) and serum creatinine (*p *< 0.001), which progressively increased from T1 to T3 groups. Inflammatory markers, such as white blood cell count, also showed a statistically significant increment (*p *= 0.027) across tertiles. Notably, previous medical history showed significant differences in diabetes mellitus prevalence (*p *= 0.005), with the T3 group displaying a substantially higher proportion of patients with prior diabetes (31.51% vs. 19.15% in the T1 group). Severity scores, including APS III and APACHE IV, demonstrated a consistent and statistically significant increase across ACAG tertiles (*p *< 0.001 for both), suggesting a potential correlation between ACAG levels and clinical severity. These findings provide a comprehensive baseline characterization of the study population, highlighting potential metabolic and physiological variations associated with ACAG tertiles. The 28-day ICU mortality rate was 13.60% (97/713) in our cohort. The 28-day mortality rate from the lowest tertile (4.75–14.25) to the highest tertile (18.10–41.50) ACAG was 18 (7.66%), 35 (14.58%), and 44 (18.49%) (Table 1). Critically, 28-day ICU mortality exhibited a significant correlation with ACAG tertiles (*p *= 0.002), suggesting a potential link between ACAG and patient outcomes.

### 3.2. Association between ACAG and 28-day mortality in patients with sepsis complicated with chronic heart failure

Table 2 details the covariate-adjusted associations between ACAG levels and 28-day mortality risk in sepsis patients with comorbid chronic heart failure, analyzed through Cox proportional risk models. Our analysis revealed a statistically significant association between Albumin Corrected Anion Gap (ACAG) and 28-day in-ICU mortality among sepsis patients with chronic heart failure. In the crude model, ACAG demonstrated a consistent hazard ratio of 1.07 (95% CI: 1.04–1.11, *p *< 0.0001), indicating a progressive mortality risk with increasing ACAG values. After stratifying ACAG into three tertiles, we observed a dose-response relationship: compared to the reference group (T1, 4.75–14.25), the second tertile (T2, 14.25–18.05) showed a marginally significant increased mortality risk (HR: 1.78, 95% CI: 1.01–3.14, *p *= 0.0473), while the highest tertile (T3, 18.10–41.50) exhibited a substantially higher mortality risk (HR: 2.17, 95% CI: 1.26–3.77, *p *= 0.0056). After comprehensive multivariable adjustment for potential confounders including demographic, clinical, and physiological parameters, the association remained robust. Model I (adjusted for age, sex, and BMI) and Model II (additionally adjusted for advanced clinical parameters) maintained the significant trend, with the highest tertile showing an adjusted hazard ratio of 2.18 (95% CI: 1.13–4.20, *p *= 0.0200). The trend test across tertiles remained statistically significant (*p *= 0.0193), suggesting a progressive mortality risk with increasing ACAG values.

**Table 2:** Association between ACAG and 28-day mortality in patients with sepsis complicated with chronic heart failure.

Outcome	Crude Model		Model Ⅰ		Model Ⅱ	
	HR (95%CI)	P-value	HR (95%CI)	P-value	HR (95%CI)	*p* value
ACAG (mmol/l)	1.07 (1.04,1.11)	<0.01	1.08 (1.04,1.12)	<0.01	1.08 (1.03,1.13)	<0.01
ACAG (tertile)						
T1 (4.75–14.25)	Reference		Reference		Reference	
T2 (14.25–18.05)	1.78 (1.01,3.14)	0.05	1.96 (1.09,3.52)	0.02	1.61 (0.84,3.08)	0.15
T3 (18.10–41.50)	2.17 (1.26,3.77)	<0.01	2.42 (1.38,4.25)	<0.01	2.18 (1.13,4.20)	0.02
*p* for trend		<0.01		<0.01		0.02

**Abbreviations:** ACAG Albumin Corrected Anion Gap; HR, hazard ratio; CI, confidence interval. Multivariable cox regression models evaluating the association between Albumin Corrected Anion Gap and 28-day in-ICU mortality. Crude model: adjusted for none. Model I adjusted for age, sex and BMI. Model II adjusted for age, sex, BMI, Temperature, Heart rate, MAP, Blood urea nitrogen, Glucose, Serum Creatinine, Serum potassium, Diabetes, AMI, Arrhythmia, acute Physiology Score III, Apache IV score.

### 3.3. The smoothing curves and Kaplan-Meier survival curve analysis

**Fig 2:**
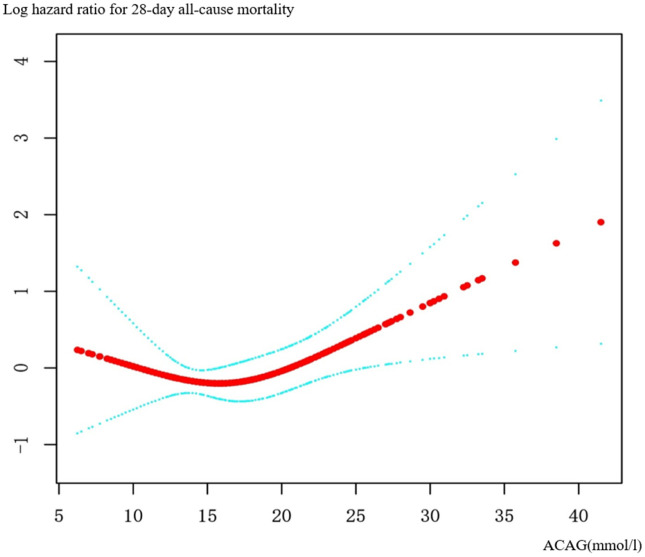
Smooth curve fitting plots. Associations between the ACAG and 28-day mortality in patients with sepsis complicated with chronic heart failure. Solid rad line represents the smooth curve fit between variables. Blue bands represent the 95% of confidence interval from the fit. Adjusted for age (years), sex, BMI, Temperature, Heart rate, MAP, Blood urea nitrogen, Glucose, Serum Creatinine, Serum potassium, Diabetes, AMI, Arrhythmia, Acute Physiology Score III, Apache IV score.

The smoothing curves (Fig 2) representation depicts the link between Albumin Corrected Anion Gap (ACAG) and ICU mortality. The analysis illustrates a clear positive correlation: as ACAG levels increase, there is a corresponding rise in 28-day mortality rates. The red line, representing the fitted smoothed curve, exhibits a linear increase, indicating that higher ACAG values are indicative of a heightened risk of death. The shaded blue regions surrounding the curve indicate the confidence intervals, which widen as ACAG values increase, suggesting greater variability in mortality outcomes at higher ACAG levels.

**Fig 3:**
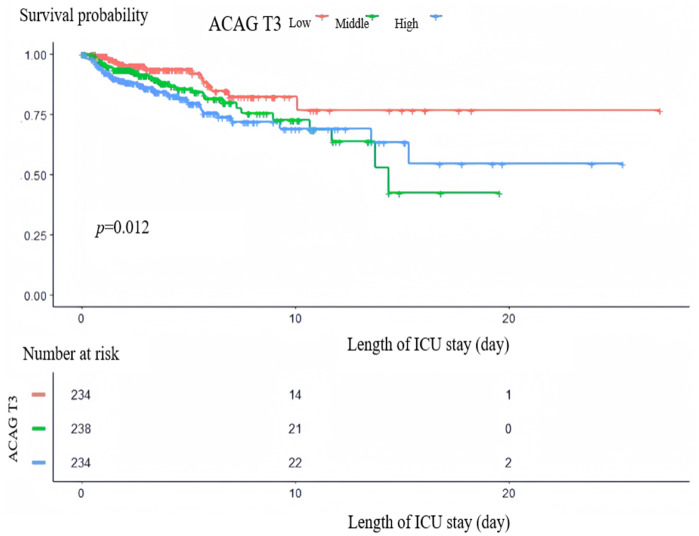
Kaplan-Meier survival curve. Kaplan-Meier survival analysis of different ACAG groups in patients with sepsis complicated with chronic heart failure.

The Kaplan-Meier survival analysis curve (Fig 3) indicated that participants with high ACAG value had a significantly higher mortality rate in comparison to those with low ACAG values ((log-rank *p *= 0.012, Fig 3).

### 3.4. Subgroup analysis

**Fig 4:**
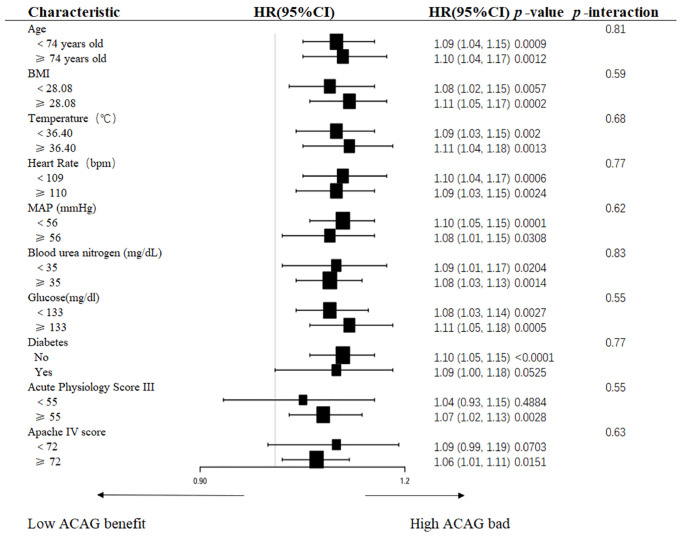
Forest plot. Forest plots of stratified analyses of ACAG and the 28-day all‑cause mortality. HR, hazard ratio; CI, confidence interval; BMI, body mass index; MAP, mean arterial pressure; AMI, acute myocardial infarction. Stratified analyses based on age, BMI, temperature, heart rate, mean arterial pressure, diabetes, blood urea nitrogen, glucose, acute physiology score III, and Apache IV score.

Through forest plot (Fig 4) visualization analysis, this study confirmed that the stability of the link between the ACAG levels and 28-day all-cause mortality in participants with sepsis complicated with chronic heart failure remained consistent across various clinical subgroups. In multivariable analyses stratified by demographic characteristics (age, BMI), physiological parameters (temperature, heart rate, mean arterial pressure), comorbidities (diabetes), laboratory markers (BUN, glucose), and critical illness severity scores (APS III, APACHE IV), no statistically significant interaction effects were observed between ACAG levels and these predefined subgroups (interaction *p*-values ranging from 0.55 to 0.83).

### 3.5. Sensitivity analyses

About the sensitivity analyses, the Cox regression models were performed on complete cases. Due to the presence of missing data in the adjustment variables of the regression model, the missing values were addressed. We used multiple imputation, based on 5 replications and a chained equation approach method in the R MI procedure, to account for missing data on age, sex, BMI, temperature, heart rate, MAP, blood urea nitrogen, glucose, serum creatinine, serum potassium, diabetes, AMI, arrhythmia, acute Physiology Score III, Apache IV score. The results of the Cox regression analysis, based on five created datasets with pooled estimates using Rubin’s rule, are presented in S1 Table. By observing ACAG and conducting multivariate Cox regression analysis on the relationship between ACAG and mortality rate, the results showed that the association between ACAG and 28-day all-cause mortality rate before and after handling missing data was consistent with the preliminary analysis, and a significant association was observed between ACAG and 28-day all-cause mortality rate.

## 4. Discussion

This retrospective study indicated that for each 1 mmol/l increment in ACAG, the risk of 28-day all-cause mortality in critically ill patients with sepsis complicated with chronic heart failure increased by 8% (adjusted HR:1.08). A statistically significant association was identified between elevated ACAG levels and 28-day all-cause mortality in such patients (adjusted HR:1.08, 95%CI: 1.03–1.13); p < 0.001), underscoring the necessity for enhanced clinical vigilance in monitoring high-risk populations. When participants were stratified into tertiles based on ACAG levels, the highest tertile (HR 2.18, p = 0.02) exhibited a significantly increased mortality rate compared to the lowest tertile, with Kaplan-Meier survival curves consistently demonstrating this association throughout the follow-up period. What’s more, subgroup analyses indicated that the stability of the link between ACAG and 28-day mortality in patients with sepsis complicated with chronic heart failure across various subgroups. Within intensive care unit (ICU) settings, both sepsis and chronic heart failure represent prevalent high-risk comorbidities. Notably, patients with pre-existing chronic heart failure who develop sepsis demonstrate substantially elevated mortality risk, with this compounded risk profile persisting throughout ICU hospitalization [20,21]. Therefore, this is the first study to examine the relationship between ACAG and acute-phase prognosis in patients with sepsis combined with chronic heart failure. Our findings suggest that high levels of ACAG may serve as a potential prognostic marker for acute-phase mortality risk in this patient population. ACAG may be an invaluable clinical decision-making tool for managing sepsis combined with chronic heart failure.

Maintaining proper acid-base homeostasis is crucial for ensuring optimal physiological processes, with imbalances potentially leading to severe clinical manifestations. Among various types of acid-base imbalances, metabolic acidosis represents the most frequently encountered type in clinical practice. This condition is conventionally classified into two distinct subtypes based on anion gap measurements: elevated anion gap (AG) metabolic acidosis and non-anion gap (normal AG) metabolic acidosis. In a prospective cohort study of coronary artery disease (CAD) patients, those with serum anion gap (AG) levels exceeding 15.92 mmol/L demonstrated a 5.17-fold increased risk of 30-day all-cause mortality, with elevated AG concentrations showing a deteriorating cardiac function [22]. Emerging evidence indicates that elevated AG may contribute to the pathogenesis of multiple cardiovascular pathologies, with accumulating clinical data demonstrating its association with poorer prognostic trajectories in patients with established cardiovascular disease through mechanisms involving acid-base imbalance and metabolic dysregulation [22,23]. Since albumin accounts for a significant portion of the unmeasured anions, fluctuations in albumin levels consequently affected the AG levels. This change may potentially impact physicians’ assessment of the accuracy of laboratory results. Research shows that albumin influences AG levels, and the accurate interpretation of AG values therefore requires correcting for albumin levels [24,25]. ICU-admitted patients typically demonstrate rapid declines in serum albumin concentrations during the acute phase, with subsequent elevations observed during clinical recovery that correlate with improvements in nutritional status [26]. Therefore, accounting for albumin levels could be essential when evaluating the association between AG and clinical outcomes, particularly in cases exhibiting abnormally low serum protein concentrations. The following conclusions confirm the above viewpoint. In a study of 12246 patients with Sepsis, ACAG has the highest predictive value for in-hospital mortality of intensive care patients with sepsis, which is better than albumin and AG. Evaluating in – hospital mortality with ACAG to inform clinical decisions may yield the maximum net benefit [27]. A Chinese cohort study revealed that, compared with the normal ACAG group, the high ACAG group had a significantly lower 1 – year cumulative survival rate (55.0% vs. 67.7%, p = 0.046), thus confirming ACAG as an independent risk factor for 1 – year mortality in sepsis patients [28]. Hu et al. indicated that in ICU patients with sepsis, an ACAG level exceeding 21.25 mmol/L predicted the risk of in-hospital mortality, showing superior predictive value compared to AG and albumin [27]. Aydin SS et al. demonstrated a significant link between elevated ACAG levels and adverse outcomes in heart failure (HF) patients, establishing this biomarker as an independent prognostic indicator for hospitalized HF populations [29,30]. Metabolic acidosis, characterized by a decrease in serum bicarbonate levels, has been associated with various detrimental effects on organ function, particularly in patients with chronic kidney disease (CKD) and heart failure (CHF) [30,31]. The presence of hypoalbuminemia, defined as low serum albumin levels, further complicates this scenario, as it has been shown to independently predict acute kidney injury (AKI) and mortality within the intensive care population [32]. The ACAG demonstrates enhanced diagnostic accuracy in identifying metabolic acidosis in patients diagnosed with hypoproteinemia, as it explains the influence of diminished serum albumin concentrations on the anion gap [33,34]. The interplay between metabolic acidosis and hypoalbuminemia is critical in exacerbating cardiac and renal dysfunction, which can lead to adverse clinical outcomes. Despite the clinical significance of ACAG in critical care settings, a paucity of relevant research evidence exists regarding the prognostic relevance of ACAG in the population. Our study investigated the correlation between the ACAG and 28‑day all‑cause mortality in these individuals on eICU database. After adjusting for confounding factors, a statistically significant association was identified between elevated ACAG levels and 28-day all-cause mortality in critically ill patients with sepsis complicated with chronic heart failure (adjusted HR:1.08, 95%CI:1.03–1.13; p < 0.001). Furthermore, the ACAG had a linear correlation with 28 all‑cause mortality in this cohort.

The mechanism underlying the link between the ACAG and sepsis combined with CHF is not fully elucidated. It may be related to the following three mechanisms. Firstly, the interplay between sepsis and chronic heart failure creates a dual metabolic burden that significantly impacts patient outcomes. In cases of sepsis, particularly acute acidemia, there is a notable increase in anion gap (AG) levels, coupled with a decrease in serum albumin (Alb) due to inflammation and malnutrition, leading to a detrimental cycle of worsening health [35,36]. This cycle is exacerbated in patients with chronic heart failure, where chronic tissue hypoperfusion contributes to renal dysfunction, further elevating AG levels through mechanisms such as uremic acidosis and protein loss [37,38]. Secondly, the interaction between organ systems is particularly pronounced in heart failure patients who often present with renal impairment. Sepsis can aggravate this renal injury, resulting in elevated AG and reduced Alb levels, which are critical markers of organ dysfunction [39,40]. The presence of renal dysfunction in heart failure patients is significant, with studies indicating that nearly 60% of chronic heart failure patients also exhibit renal impairment, which complicates their clinical management [37,41]. Third, the immune metabolic regulation during sepsis is influenced by the baseline inflammatory state present in chronic heart failure. This pre-existing inflammation can intensify the cytokine storm characteristic of sepsis, leading to further cellular damage and organ failure [36,42]. Low Alb levels, often seen in these patients, diminish antioxidant defenses, thereby accelerating the progression to multiple organ failure [43,44]. The complexity of these interactions underscores the critical need for integrated management strategies in patients suffering from both conditions to mitigate the risk of severe outcomes [45].

Our findings corroborate previous studies that have linked anion gap metabolic acidosis (ACAG) to increased mortality in sepsis patients [46,47]. Prior research has established a connection between acid-base disturbances and adverse outcomes in critically ill patients, our research extends the implications of ACAG beyond sepsis, highlighting its relevance in populations with congestive heart failure (CHF). The implications of these findings are critical, as metabolic acidosis is a common complication in CHF, often resulting from ischemia and hypoxia due to hemodynamic instability and the effects of diuretics [48,49]. The relationship between elevated ACAG and increased mortality risk underscores the need for careful monitoring of acid-base status in this population. In CHF patients, the presence of metabolic acidosis can signify a more severe underlying pathology, as it often correlates with worse outcomes and higher mortality rates [50]. The combination of metabolic acidosis and hypoalbuminemia may create a vicious cycle, where each condition exacerbates the other, leading to a significant decline in patient prognosis [51]. The study aligns with previous literature that highlights the role of acid-base imbalances in the prognosis of critically ill patients, suggesting that interventions aimed at correcting these disturbances could potentially improve outcomes [49]. This relationship underscores the necessity for vigilant monitoring and management of acid-base status and serum albumin levels in these vulnerable populations. This reinforces the potential of ACAG as a valuable tool in the management of sepsis and CHF patients, enabling clinicians to make more informed decisions regarding treatment strategies. Our study parallels published literature in demonstrating the relationship between mortality and ACAG levels.

## 5. Conclusion and limitations

To sum up, elevated ACAG is positively associated with increased 28-day all‑cause mortality in patients with sepsis complicated with chronic heart failure. Routine ACAG assessment may enhance risk stratification and guide personalized interventions. However, it is important to acknowledge the limitations of our study. Firstly, this study relies on the eICU database, which has a comparatively small sample size, and the results may not be indicative of the whole population. Whether the study results can be extrapolated to a broader population remains to be further explored. We expect further studies with larger scale may consolidate our results. Secondly, as a secondary analysis of prospectively collected observational cohort data, this study’s non-interventional design inherently precludes causal inference due to potential residual confounding and the absence of randomized allocation. the observed ACAG-mortality correlation may be subject to residual confounding from unobserved variables not captured in our multivariable models. Our analysis encountered incomplete data records for selected covariates. Future studies should aim to validate these results through largescale prospective research and explore the dynamic changes in ACAG over time. Understanding these dynamics could lead to improved therapeutic strategies aimed at mitigating the adverse effects of these metabolic disturbances on cardiac function. Third, the eICU database lack of information on interventions (such as the medication situation) during initial stabilization may have influenced ACAG levels and survival. Lastly, it should be noted that the geographic limitations of this study merit consideration. Given that the investigation was exclusively conducted within U.S. healthcare systems, the generalizability of findings to international intensive care units may be constrained by variations in clinical protocols or resource availability across different healthcare settings.

## Supporting information

S1 Table Cox regression models for the association between ACAG and 28-day mortality in patients with sepsis complicated with chronic heart failure after handling missing data in all adjustment variables. (DOCX)
