# Supplementary material for: Link between the albumin-corrected anion gap and 28 day all‑cause mortality among patients with sepsis complicated with chronic heart failure: A retrospective analysis using the eICU Collaborative Research Database
Source: PLoS One. 2025 Dec 16;20(12):e0337973. doi: 10.1371/journal.pone.0337973 (PMC12707619; doi:10.1371/journal.pone.0337973)
Supplement: S1 Table — (DOCX) [file pone.0337973.s001.docx]

**Supplementary material**

**Table S1** Cox regression models for the association between ACAG and 28-day mortality in patients with sepsis complicated with chronic heart failure after handling missing data in all adjustment variables

| Outcome | Crude Model | |  | Model Ⅰ | |  | Model Ⅱ | |
| --- | --- | --- | --- | --- | --- | --- | --- | --- |
|  | HR (95%CI) | P-value |  | HR (95%CI) | P-value |  | HR (95%CI) | *p* value |
| ACAG(mmol/l) | 1.07 (1.04,1.11) | <0.01 |  | 1.08 (1.04,1.12) | <0.01 |  | 1.07 (1.03,1.11) | <0.01 |
| ACAG(tertile) |  |  |  |  |  |  |  |  |
| T1(4.75-14.25) | Reference |  |  | Reference |  |  | Reference |  |
| T2(14.25-18.05) | 1.78(1.01,3.14) | 0.05 |  | 1.87(1.05,3.31) | 0.02 |  | 1.69(0.95,3.03) | 0.15 |
| T3(18.10-41.50) | 2.17(1.26,3.77) | <0.01 |  | 3.00(1.33,4.00) | <0.01 |  | 1.97 (1.09,3.57) | 0.0200 |
| *p* for trend |  | <0.01 |  |  | <0.01 |  |  | 0.02 |

**Abbreviations:** Ref, Reference; ACAG Albumin Corrected Anion Gap；HR, hazard ratio；CI, confidence interval. Multivariable cox regression models evaluating the association between Albumin Corrected Anion Gap and 28-day in-ICU mortality. Crude model: adjusted for none.

**Model I** adjusted for age，sex and BMI.

**Model II** adjusted for age，sex，BMI，Temperature，Heart rate，MAP，Blood urea nitrogen，Glucose，Serum Creatinine，Serum potassium，Diabetes ，AMI，Arrhythmia, acute Physiology Score III，Apache IV score.
